# Predictive Nomogram and Risk Factors for Lymph Node Metastasis in Bladder Cancer

**DOI:** 10.3389/fonc.2021.690324

**Published:** 2021-06-16

**Authors:** Zijian Tian, Lingfeng Meng, Xin Wang, Tongxiang Diao, Maolin Hu, Miao Wang, Yaqun Zhang, Ming Liu

**Affiliations:** ^1^ Department of Urology, Beijing Hospital, National Center of Gerontology, Institute of Geriatric Medicine, Chinese Academy of Medical Sciences, Beijing, China; ^2^ Graduate School of Peking Union Medical College, Chinese Academy of Medical Sciences, Beijing, China

**Keywords:** bladder cancer, lymph node metastasis, prognosis, risk, nomogram

## Abstract

Lymph node metastasis (LNM) is an important prognostic factor for bladder cancer (BCA) and determines the treatment strategy. This study aimed to determine related clinicopathological factors of LNM and analyze the prognosis of BCA. A total of 10,653 eligible patients with BCA were randomly divided into training or verification sets using the 2004–2015 data of the Surveillance, Epidemiology, and End Results database. To identify prognostic factors for the overall survival of BCA, we utilized the Cox proportional hazard model. Independent risk factors for LNM were evaluated *via* logistic regression analysis. T-stage, tumor grade, patient age and tumor size were identified as independent risk factors for LNM and were used to develop the LNM nomogram. The Kaplan-Meier method and competitive risk analyses were applied to establish the influence of lymph node status on BCA prognosis. The accuracy of LNM nomogram was evaluated in the training and verification sets. The areas under the receiver operating characteristic curve (AUC) showed an effective predictive accuracy of the nomogram in both the training (AUC: 0.690) and verification (AUC: 0.704) sets. In addition, the calibration curve indicated good consistency between the prediction of deviation correction and the ideal reference line. The decision curve analysis showed that the nomogram had a high clinical application value. In conclusion, our nomogram displayed high accuracy and reliability in predicting LNM. This could assist the selection of the optimal treatment for patients.

## Introduction

Bladder cancer (BCA) is one of the most common cancers of the urinary system, with a high mortality and morbidity rate worldwide. In 2020, there were about 81,400 new cases of BCA in the USA, with approximately 17,980 deaths (1). The presence of lymph node metastasis (LNM) in patients with BCA is one of the most useful markers of tumor invasiveness, and up to 25% of muscle-invasive BCA patients and 8% non-muscle-invasive BCA patients were reported to have LNM (2). Among patients who received treatment for BCA, the 5-year overall survival (OS) rate of patients with negative lymph nodes was 39% to 56%, whereas that of patients with positive lymph nodes was 30% to 32% (3–6). Positive lymph nodes have also been proven to be associated with BCA recurrence and cancer-specific death (5, 7, 8). The treatment strategy for patients with BCA varies according to the lymph node status. BCA patients with LNM can still get cured before distant metastasis if appropriate treatment is chosen (9). Therefore, it is crucial to accurately estimate the lymph node status of patients with BCA.

The role of nomograms in predicting LNM of BCA has been extensively studied. Karakiewicz et al. (10) assessed a multicenter cohort of 726 patients, but the maximum accuracy of the predictive nomogram for LNM was only 63.1%, which means that 36.9% of patients were misclassified. Moreover, when the performance of the nomogram was verified in other studies, it was found to be considerably reduced (11, 12), showing clinical ineffectiveness in the analysis of decision curve analysis (DCA) (13). The two other nomograms for predicting LNM in BCA are based on multivariate analyses, but the studies were conducted in a single institutional center with a limited sample size; thus, the selection bias is considerable (14, 15). To our knowledge, no large-scale multicenter study has been performed to formulate a quantitative prediction nomogram.

To this end, we carried out this study using clinical, pathological, and demographic information contained in the Surveillance, Epidemiology, and End Results (SEER) database to identify risk factors for LNM of BCA and construct a nomogram for predicting the incidence of LNM in BCA.

## Materials and Methods

### Patients

The SEER database is a cancer-specific database in the United States that contains the morbidity, mortality, and illness of millions of patients with malignant tumors. The inclusion criteria were a pathological diagnosis of BCA between 2004 and 2015, undergoing surgery, transitional cell carcinoma as pathological type, and involvement of at least one lymph node. The exclusion criteria were age <18 years, distant metastasis, receiving preoperative radiotherapy (to exclude its influence on LNM), and incomplete clinicopathological data. The entire data set of the SEER database was randomly divided into a training set and a verification set using a ratio of 1:1. The SEER database is a public database, and we have provided a signed SEER research data agreement form to the SEER project, which granted access to and analysis of SEER data; thus, informed consent is not required.

### Construction and Validation of the Nomogram

Univariate and multivariate analyses were performed on patients with BCA in the SEER cohort to evaluate the independent risk factors and prognostic factors. The logistic regression model was utilized to identify risk factors for LNM. The Cox proportional hazards model was used to determine potentially important prognostic factors of BCA. Furthermore, based on the logistic regression model plus Cox proportional hazards model, nomograms of LNM and OS were established. Meanwhile, the accuracy of the nomogram was evaluated using a calibration curve in the training and verification sets. A receiver operating characteristic (ROC) curve was plotted, and we calculated the area under the curve (AUC) to quantify the discriminatory ability of the nomogram. The net benefit under each risk threshold probability was calculated *via* DCA to demonstrate the clinical application value of the nomogram. In addition, the clinical impact curve was plotted to elucidate the potential benefits of the nomogram in clinical practice.

### Statistical Analysis

All statistical analyses were accomplished by SPSS version 25 (IBM Corp., Armonk, NY, USA) and R version 3.6.1 (The R Project, Vienna, Austria). Categorical variables are evaluated by the chi-square test. Univariate and multivariate logistic regression analyses and Cox regression models were utilized to screen the risk factors and prognostic factors. The corresponding software packages (rms, foreign, survival ROC, rmda, survival, cmprsk, ggplotify, magick, survminer, cowplot, and stdca) of R version 3.6.1 were used to construct the nomogram, calibration curve, ROC curve, Kaplan-Meier curve, competitive risk curve, DCA, and clinical impact curve. All statistical tests were bilateral, and a P-value <0.05 was considered significant.

## Results

### Demographics and Pathological Characteristics

A total of 10,653 patients with BCA from the SEER database who met the criteria were registered in the present study. A total of 5,327 patients, including 2,757 patients with positive lymph nodes and 2,570 patients with negative lymph nodes, were randomly assigned to a training set, and the remaining 5,326 patients, including 2,768 patients with positive lymph nodes and 2,558 patients with negative lymph nodes, were assigned to the verification set. The characteristics of these patients are presented in **Table 1**.

**Table 1:** Clinicopathological characteristics of the cohort by lymph node status.

Characteristics	Training cohort			Validation cohort		
	LNM (-)	LNM (+)	P value	LNM (-)	LNM (+)	P value
	N=2570	N=2757		N=2558	N=2768	
**Age**			<0.001			<0.001
<50	89 (3.5%)	149 (5.4%)		101 (3.9%)	135 (4.9%)	
50-65	719 (28.0%)	993 (36.0%)		662 (25.9%)	987 (35.7%)	
65-79	1285 (50.0%)	1320 (47.9%)		1318 (51.5%)	1336 (48.3%)	
>80	477 (18.6%)	295 (10.7%)		477 (18.6%)	310 (11.2%)	
**Sex**			0.135			0.834
Female	609 (23.7%)	702 (25.5%)		635 (24.8%)	694 (25.1%)	
Male	1961 (76.3%)	2055 (74.5%)		1923 (75.2%)	2074 (74.9%)	
**Race**			0.303			0.896
Caucasians	2253 (87.7%)	2417 (87.7%)		2256 (88.2%)	2457 (88.8%)	
Afro-Americans	153 (6.0%)	185 (6.7%)		154 (6.0%)	154 (5.6%)	
Other	156 (6.1%)	151 (5.5%)		143 (5.6%)	151 (5.5%)	
Unknown	8 (0.3%)	4 (0.1%)		5 (0.2%)	6 (0.2%)	
**Grade**			<0.001			<0.001
Grade I	74 (2.9%)	10 (0.4%)		52 (2.0%)	10 (0.4%)	
Grade II	148 (5.8%)	47 (1.7%)		151 (5.9%)	63 (2.3%)	
Grade III	747 (29.1%)	774 (28.1%)		790 (30.9%)	724(26.2%)	
Grade IV	1601 (62.3%)	1926 (69.9%)		1565 (61.2%)	1971 (71.2%)	
**Tumor size**			<0.001			<0.001
<1cm	165 (6.4%)	81 (2.9%)		136 (5.3%)	71 (2.6%)	
1-2cm	292 (11.4%)	213 (7.7%)		312 (12.2%)	236 (8.5%)	
2-3cm	457 (17.8%)	439 (15.9%)		450 (17.6%)	456 (16.5%)	
3-4cm	480 (18.7%)	522 (18.9%)		473 (18.5%)	524 (18.9%)	
4+cm	1176 (45.8%)	1502 (54.5%)		1187 (46.4%)	1481 (53.5%)	
**T**			<0.001			<0.001
T1	372 (14.5%)	113 (4.1%)		425 (16.6%)	113 (4.1%)	
T2	1004 (39.1%)	895 (32.5%)		1049 (41.0%)	909 (32.8%)	
T3	783 (30.5%)	1142 (41.4%)		737 (28.8%)	1157 (41.8%)	
T4	246 (9.6%)	585 (21.2%)		189 (7.4%)	565 (20.4%)	
Ta	148 (5.8%)	21 (0.8%)		142 (5.6%)	20 (0.7%)	
Tis	17 (0.7%)	1 (0.1%)		16 (0.6%)	4 (0.1%)	

LNM, lymph node metastasis.

### Prognostic Factors for BCA and Construction of the Nomogram

The Cox regression model was utilized to verify the statistical effects of the clinicopathological factors (**Supplementary Table 1**). According to the results of the univariate Cox regression analysis of the training set, six factors were significantly related to BCA prognosis, namely age, race, tumor grade, tumor size, T-stage, and N-stage. We included all these significant factors in the multivariate Cox analysis. The analysis showed that age, race, tumor grade, tumor size, T-stage, and N-stage were independent predictive parameters related to BCA prognosis. On this basis, the OS nomogram was plotted (**Supplementary Figure 1**).

To reduce bias, we analyzed the above factors in BCA patients without LNM. The results showed that age, tumor grade, tumor size, and T-stage are independent risk factors related to prognosis (**Supplementary Table 2**).

In this study, a calibration curve, which is the best method to intuitively compare the consistency between the predicted risk and the absolute risk, is given *via* the bootstrap resampling method (16). The calibration curves of both the training (**Supplementary Figures 2A, B**) and verification (**Supplementary Figures 3A, B**) sets lie on the 45° line, reflecting an excellent absolute risk estimate. Further, the AUCs of the training and verification sets were 0.697 and 0.702, respectively, indicating good consistency and reliability in estimating BCA prognosis (**Supplementary Figure 2C**). Finally, the DCA curves of the training (**Supplementary Figure 2D**) and validation (**Supplementary Figure 3C**) sets showed the clinical usefulness of the prognostic nomogram.

### Independent Risk Factors for LNM and the Development of a Nomogram

We used univariate and multivariate logistic regression analyses to determine the independent risk factors for LNM. These factors included age, tumor grade, tumor size, and T-stage (**Table 2**). Concerning age, the risk of developing LNM was lower in older patients, especially in patients aged >80 years, than in younger patients (OR=0.288, 95%CI: 0.209–0.397, P<0.001). Further, tumor grade was shown to be an important independent predictor; LNM was more likely to develop in undifferentiated cancer than well-differentiated cancer (OR=3.730, 95%CI: 1.831–7.599, P<0.001). In addition, large tumors were more likely to develop LNM than small tumors (>4 cm *vs.* <1 cm, OR=1.839, 95%CI: 1.367–2.473, P<0.001). Regarding T-stage, the risk of LNM was the highest in T4 tumors (OR=7.587, 95%CI: 5.824–9.883, P<0.001).

**Table 2:** Logistic regression analysis of the risk factors for lymph nodes metastasis.

Clinicopathological variables	Univariate analysis		Multivariate analysis	
	OR (95%CI)	P value	OR (95%CI)	P value
Age at diagnosis				
<50	Reference		Reference	
50-65	0.825 (0.624-1.091)	0.177	0.760 (0.564-1.024)	0.071
65-79	0.614 (0.467-0.807)	<0.001	0.541 (0.404-0.725)	<0.001
>80	0.369 (0.274-0.499)	<0.001	0.288 (0.209-0.397)	<0.001
Sex				
Female	Reference			
Male	0.909 (0.802-1.030)	0.135		
Race				
Caucasians	Reference			
Afro-Americans	1.127 (0.903-1.407)	0.290		
Other	0.902 (0.716-1.137)	0.383		
Unknown	0.466 (0.140-1.550)	0.213		
Grade				
Grade I	Reference		Reference	
Grade II	2.350 (1.124-4.913)	0.023	1.687 (0.773-3.681)	0.189
Grade III	7.667 (3.932-14.953)	<0.001	3.063 (1.497-6.269)	0.002
Grade IV	8.902 (4.584-17.287)	<0.001	3.730 (1.831-7.599)	<0.001
Tumor size				
<1cm	Reference		Reference	
1-2cm	1.486 (1.080-2.045)	0.015	1.290 (0.919-1.812)	0.141
2-3cm	1.957 (1.455-2.632)	<0.001	1.542 (1.124-2.115)	0.007
3-4cm	2.215 (1.652-2.971)	<0.001	1.662 (1.215-2.272)	0.001
4+cm	2.602 (1.973-3.431)	<0.001	1.839 (1.367-2.473)	<0.001
T				
T1	Reference		Reference	
T2	2.935 (2.334-3.690)	<0.001	2.849 (2.256-3.598)	<0.001
T3	4.801 (3.817-6.039)	<0.001	4.798 (3.791-6.073)	<0.001
T4	7.829 (6.049-10.132)	<0.001	7.587 (5.824-9.883)	<0.001
Ta	0.467 (0.282-0.773)	0.003	0.745 (0.438-1.268)	0.278
Tis	0.194 (0.025-1.471)	0.113	0.299 (0.038-2.321)	0.248

OR, odd ratio; 95%CI, 95% confidence intervals.

**Figure 1:**
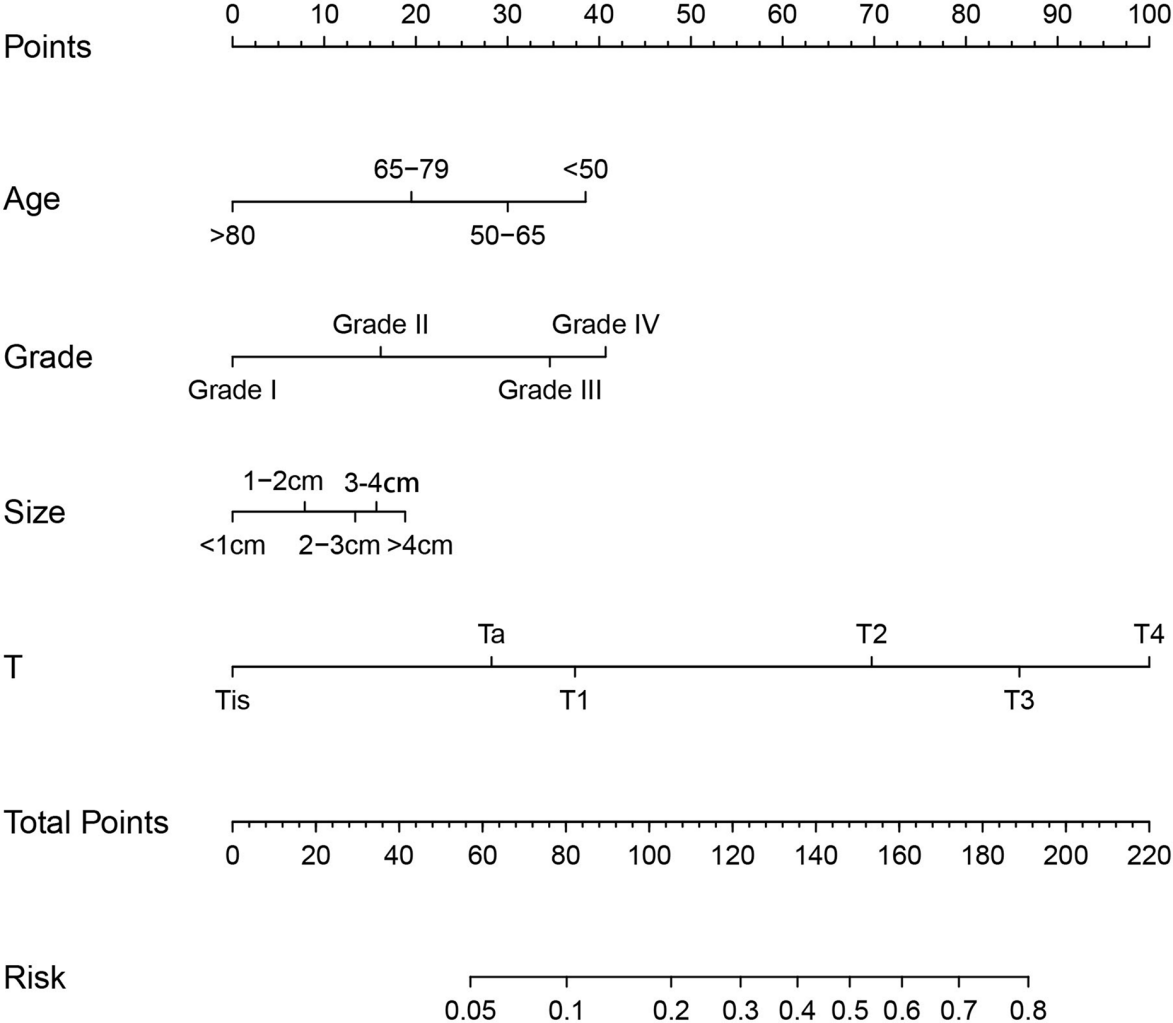
Nomogram for predicting lymph node metastasis in patients with bladder cancer.

**Figure 2:**
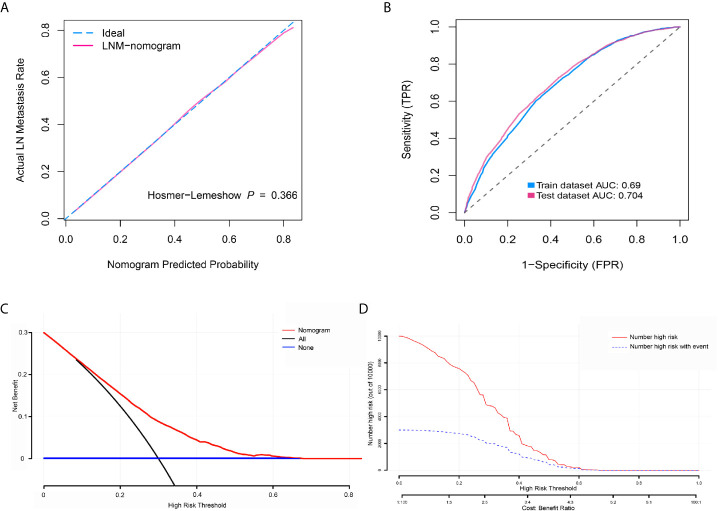
Calibration curve, receiver operating characteristic curve, decision curve analysis (DCA), and clinical impact curve for predicting lymph node metastasis (LNM) in patients with bladder cancer (BCA). **(A)** Calibration curve of positive lymph node probability nomogram in the training set (bootstrap method, 1000 repetitions). **(B)** Area under the curve for predicting the LNM of patients with BCA in the training and verification sets. **(C)** The DCA curve of the training set. The x-axis and y-axis mean the threshold probability and net benefit, respectively. The black line indicates all patients experienced LNM, and the blue line indicates no patient developed LNM. **(D)** Clinical impact curve of the training set. The red curve demonstrates the number of people who were classified positive by the nomogram under each threshold probability, and the blue curve indicates the number of true positives under each threshold probability.

**Figure 3:**
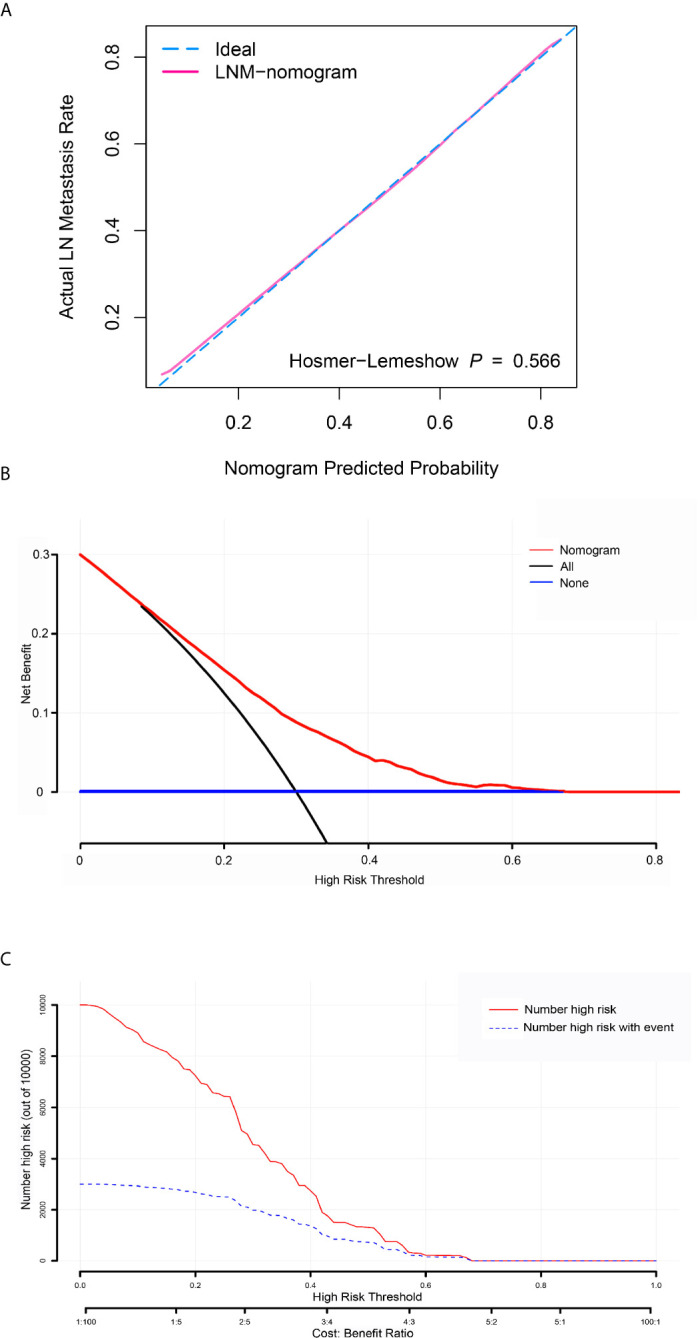
Calibration curve, decision curve analysis (DCA), and clinical impact curve for predicting lymph node metastasis (LNM) in patients with bladder cancer. **(A)** Calibration curve of positive lymph node probability nomogram in the verification set (bootstrap method, 1000 repetitions). **(B)** The DCA curve of the verification set. The x-axis and y-axis mean the threshold probability and net benefit, respectively. The black line indicates all patients experienced LNM, and the blue line indicates no patient developed LNM. **(C)** Clinical impact curve of the verification set. The red curve demonstrates the number of people classified as positive by the nomogram under each threshold probability, and the blue curve is the number of true positives under each threshold probability.

To determine the risk factors for LNM, we established a nomogram (**Figure 1**). In the LNM nomogram, T-stage contributed to the largest proportion, followed by tumor grade, age, and tumor size. The P-values of the calibration curve for the training (**Figure 2A**) and verification (**Figure 3A**) sets were calculated as 0.366 and 0.566, respectively, using the Hosmer-Lemeshow goodness-of-fit test. Moreover, the calibration curve of the nomogram was highly consistent with that of the standard curve, which demonstrates that the nomogram has a good fitting effect and is repeatable and reliable. In addition, the AUC showed an effective predictive accuracy of the nomogram in both the training (AUC: 0.690) and verification (AUC: 0.704) sets (**Figure 2B**). The clinical impact curve shows the number of BCA patients with LNM classified by the nomogram and the number of BCA patients with LNM in the original data. The results in the training (**Figures 2C, D**) and verification (**Figures 3B, C**) data sets suggest that the nomogram has good clinical application value.

### Survival Analyses Based on the Kaplan-Meier Method and Fine and Gray Model Analysis

**Figure 4:**
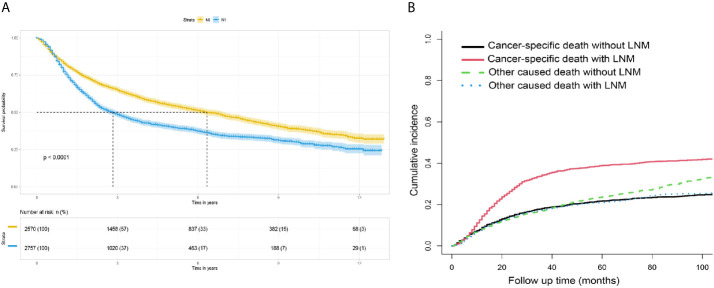
**(A)** Survival analysis: Kaplan-Meier survival curves grouped according to lymph node status in the training set. **(B)** Competitive risk curve: death from non-bladder cancer in the training set was regarded as a competitive risk event.

**Figure 5:**
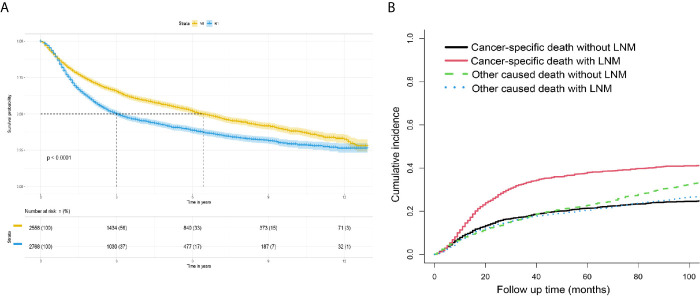
**(A)** Survival analysis: Kaplan-Meier survival curves grouped according to the lymph node status in the verification set. **(B)** Competitive risk curve: death from a non-bladder cancer cause in the verification set was regarded as a competitive risk event.

The survival curves for the groups of patients with positive and negative lymph nodes were plotted using the Kaplan-Meier method. Survival analysis indicated a significant difference between the training (P<0.001, **Figure 4A**) and verification (P<0.001, **Figure 5A**) sets. In addition, death from other causes was regarded as a competing risk event. We used the Fine and Gray competitive risk analysis in both the training (P<0.001, **Figure 4B**) and verification (P<0.001, **Figure 5B**) sets to further analyze the effect of lymph node status on BCA prognosis. The result showed that LNM was significantly associated with cancer-specific death.

## Discussion

LNM is considered the most important pathway of BCA metastasis. In addition, studies have suggested that the life expectancy of patients with BCA is determined by the extent of metastasis, which begins with cancer cells entering the lymph nodes through the lymphatic system and finally entering distant organs from the blood vessels (17). Although previous studies have confirmed that the risk of LNM in BCA is related to tumor stage and grade (18, 19), the clinical stages in 42–48% of patients were underestimated (10, 20). Moreover, it has been reported that 24% of the patients of Ta and Tis had incorrect pathological stages and were defined as LNM through follow-up inspections (10). Clinically, the prognoses of patients with the same tumor stage and grade are different; thus, predicting LNM based on the tumor stage and grade is inadequate. Therefore, enhancing other important prognostic indicators may lead to a considerable improvement in the risk stratification of patients.

Nomograms are currently one of the most widely used prediction tools due to their ability to combine clinical characteristics to generate individual probabilities of clinical events. Nomograms can also present simple statistical analysis and visualization results, which are helpful for clinical decision-making and for promoting the development of personalized medical therapy. Recently, nomograms have been broadly applied to predict the risk of LNM in different tumors and have been proven to be effective (21–23).

In this study, T-stage, tumor grade, tumor size and patient age were independent factors for LNM occurrence in BCA. T-stage was the most significant factor, and the risk of LNM was significantly higher in muscle-invasive BCA than in non-muscle-invasive BCA. Similar to our results, in a multicenter study that included 726 BCA patients, it was found that T staging was closely related to lymph node metastasis of BCA (10). In Ta-Tis BCA patients, at least 6 lymph nodes need to be removed to achieve 90% confidence that the patient is node-negative. On the contrary, for T1 patients, at least 10 lymph nodes need to be removed to ensure a 90% probability of determining the true nodular state. In patients with T2 BCA, the result of removing 25 lymph nodes is that the probability of determining the true lymph node status is >90%. In T3-4 BCA, even if it touches 30 lymph nodes, it only reaches 79.7% of the predicted value. This also confirms the importance of T staging for lymph node prediction and the correlation between T staging and lymph node status (24). Our research found that tumor grade was another important factor; undifferentiated tumors were more likely to develop LNM than well-differentiated tumors. This is the same as a previous study that included 424 patients with BCA and found that high-grade BCA patients are more likely to develop lymph node metastasis than low-grade BCA patients (18). In our study, tumor size was one of the risk factors for LNM, which is consistent with the findings of previous studies (25). The study by Xie et al. also pointed out that as the tumor size increases, the probability of positive lymph nodes also increases (15). Our study also suggests that age is a predictor of LNM; previous studies have reported that for every 10-year increase in age, the LNM of patients with BCA decreases by approximately 20% (26). In another study involving 15,624 patients with BCA, younger patients had a higher risk of LNM (27). Lymph nodes change with age (28). Older patients have a lesser lymph node cortex and medulla due to degenerative changes than young patients, leading to further lymph node degeneration into inactive forms. The presence of inactive lymph nodes eventually leads to decreased lymph flow to lymph nodes and lymph node retraction (29, 30). This mechanism may explain the effect of age on LNM.

The LNM nomogram can determine the extent of lymph node dissection intraoperatively and optimize the outcome of patients. Radical cystectomy plus pelvic lymph node dissection (PLND) is regarded as the gold standard for treating muscle-invasive BCA (31). However, the indication for standard or extended PLND has not been established. Some studies have shown that extended PLND significantly improves prognosis than standard PLND (32, 33). Dhar et al. (34) retrospectively compared 658 patients with BCA who underwent either standard or extended PLND. In the study, among patients with positive lymph nodes, the 5-year recurrence-free survival rate of those who underwent extended PLND was significantly improved (35% *vs.* 7%). However, in a recent prospective multicenter phase III clinical study that included 401 patients with BCA (standard PLND group: 203 patients; extended PLND group: 198 patients), no significant difference was found in recurrence-free survival, cancer-specific survival, and OS rates (35). Furthermore, some studies have shown that extended PLND, compared to standard PLND, does not increase the rates of disease-free survival, cancer-specific survival, and OS in patients with negative lymph nodes (32, 36). Therefore, our nomogram can be used to evaluate the risk of LNM in selecting the scope of lymph node dissection. For patients who may have LNM, choosing extended PLND suggests adequate micrometastatic lymphadenectomy and more favorable clinical results (37). However, for patients in whom negative lymph nodes were detected after evaluation using a nomogram, standard PLND, rather than extended PLND, should be selected after comprehensive consideration to avoid prolonged operation time and the risk of complications, such as autonomic nerve and ureteral injuries, lymphoceles of Clavien grade 3 or above, increased bleeding, severe nutritional and immune system problems postoperatively, and significantly prolonged hospitalization (35, 38–41).

A constant challenge for clinicians is determining the best way to combine the existing prognostic information and anatomical staging for individual prognostic assessment. Nomograms can help determine whether a patient is suitable for bladder-sparing approaches. Multimodal and trimodal therapies are effective alternatives for patients ineligible or unwilling to undergo radical cystectomy (42). It has been reported that the prognosis of bladder-sparing approaches is similar to that of radical cystectomy, and the postoperative quality of life of patients has significantly improved by the preservation of the bladder (43–45). The treatment can only be effective by choosing the appropriate treatment according to the instructions in the guidelines; hence, the choice of bladder-sparing approaches should have strict indications. Bladder-sparing approaches are not recommended for patients with BCA with high-risk features, such as LNM (42). Therefore, in patients with BCA who have not undergone lymph node dissection, our nomogram would be of great value because it can accurately evaluate the lymph node involvement and determine whether bladder-sparing approaches are appropriate.

At present, there is limited information in the literature concerning neoadjuvant therapy for patients with LNM, but in a study involving 1,739 patients with BCA with LNM, preoperative neoadjuvant chemotherapy was associated with a greater improvement of OS than radical cystectomy alone (hazard ratio=0.80, 95% CI: 0.66–0.97) (3). A study by Darwish et al. also reported that among cT2-4N1-3M0 patients who received neoadjuvant therapy, the disease category in 53 patients (12.7%) was down-staged to pT0N0, with a 72% lower risk of death and a 5-year OS of 85.4%, compared with those who remained at stage pT0N+ (9). Therefore, it is crucial to determine the lymph node status of patients with BCA before formulating the treatment strategy because although patients with LNM have a high risk of distant metastasis, they can be cured if a comprehensive multimodal treatment is actively chosen.

Our research has some limitations. First, the observational and retrospective study design allows for the existence of confounding factors. Second, there was a lack of important treatment information. The SEER database does not contain information on neoadjuvant and adjuvant chemotherapy that may affect oncological outcomes. Third, it is unclear whether adding a comprehensive treatment strategy to our study would have improved the results. Finally, this study was developed from the SEER database, and the verification did not include external data; thus, our findings may only apply to the SEER registration areas.

In conclusion, based on the clinicopathological information in our large population database, we plotted the nomogram that could predict LNM in BCA patients. After verifying the performance of the nomogram using various methods, the nomogram displayed high accuracy and reliability in predicting LNM. Thus, we believe that our nomogram can help clinicians to provide personalized treatment plans for patients.

## Data Availability Statement

The datasets presented in this study can be found in online repositories. The names of the repository/repositories and accession number(s) can be found in the article/**Supplementary Material**.

## Author Contributions

Study concept & design: ZT and ML. Data acquisition or data analysis/interpretation: LM and YZ. Literature research: XW, MW, TD, and MH. All authors contributed to the article and approved the submitted version.

## Funding

This study was financially supported by the Beijing Municipal Science and Technology Project (Z201100005620007), the Beijing Hospital Clinical Research 121 Project (BJ-2018-090) and Beijing Dongcheng District Outstanding Talent Funding Project (BJ-2020-047).

## Conflict of Interest

The authors declare that the research was conducted in the absence of any commercial or financial relationships that could be construed as a potential conflict of interest.

## Supplementary Material

Supplementary Figure 1 Nomogram for predicting overall survival in patients with bladder cancer.

Supplementary Figure 2 Calibration curve, receiver operating characteristic curve, and decision curve analysis (DCA) for predicting overall survival (OS). Calibration curve of the 3- **(A)** and 5-year **(B)** OS nomograms in the training set (bootstrap method, 1000 repetitions). **(C)** Area under the curve for predicting the OS of patients with BCA in the training and validation sets. **(D)** The DCA curve of the training set. The x-axis represents the threshold probability, and the y-axis represents the net benefit. The black line indicates that all patients are at low risk, whereas the green line indicates that all patients are at high risk.

Supplementary Figure 3 Calibration curve and decision curve analysis (DCA) for predicting overall survival in patients with bladder cancer. Calibration curve of the 3- **(A)** and 5-year **(B)** overall survival nomograms in the verification set (bootstrap method, 1000 repetitions). **(C)** The DCA curve of the verification set. The x-axis and y-axis mean the threshold probability and net benefit, respectively. The black line indicates that all patients are at low risk, whereas the green line indicates that all patients are at high risk.
