# Supplementary figures and images for: Predictive Nomogram and Risk Factors for Lymph Node Metastasis in Bladder Cancer
Source: Front Oncol. 2021 Jun 16;11:690324. doi: 10.3389/fonc.2021.690324 (PMC8242250; doi:10.3389/fonc.2021.690324)

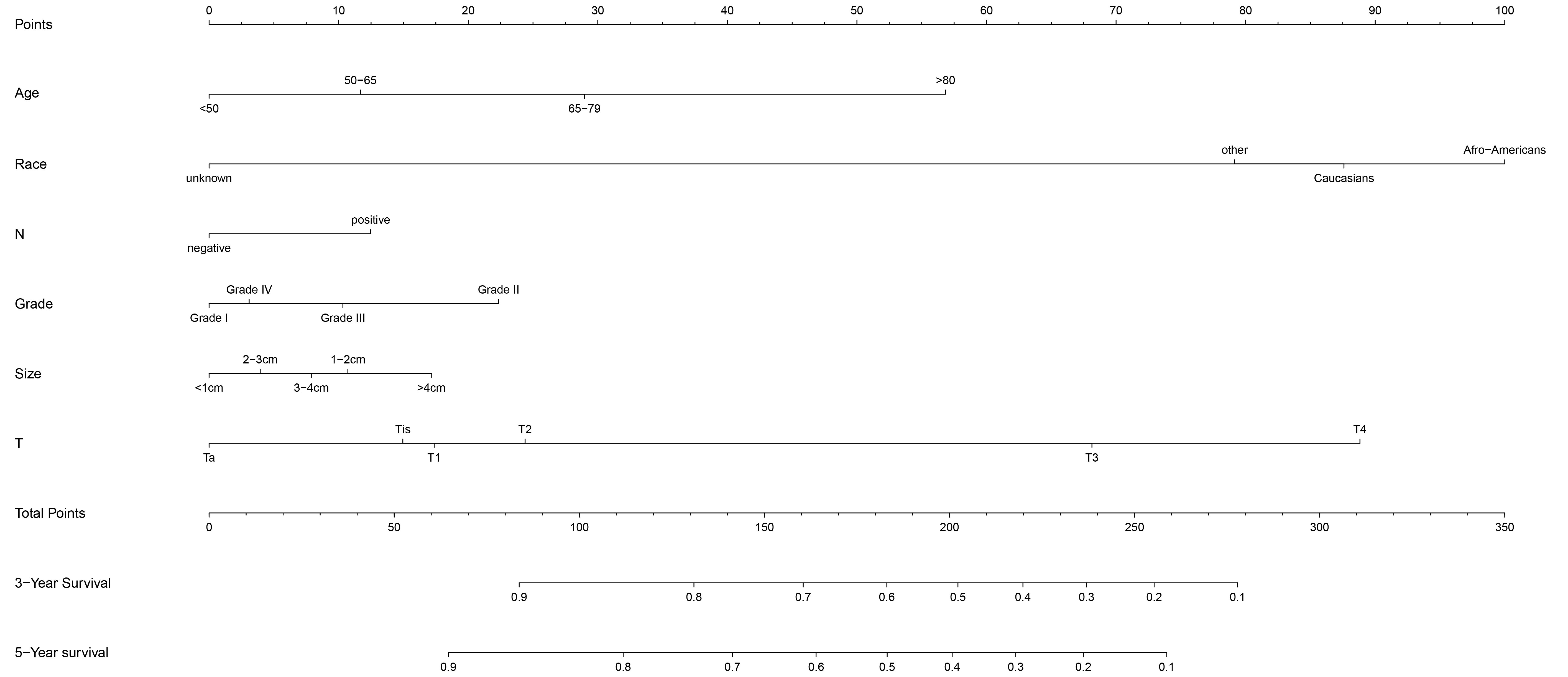

Supplement: Supplementary Figure 1 — Nomogram for predicting overall survival in patients with bladder cancer. [file Image_1.jpeg]

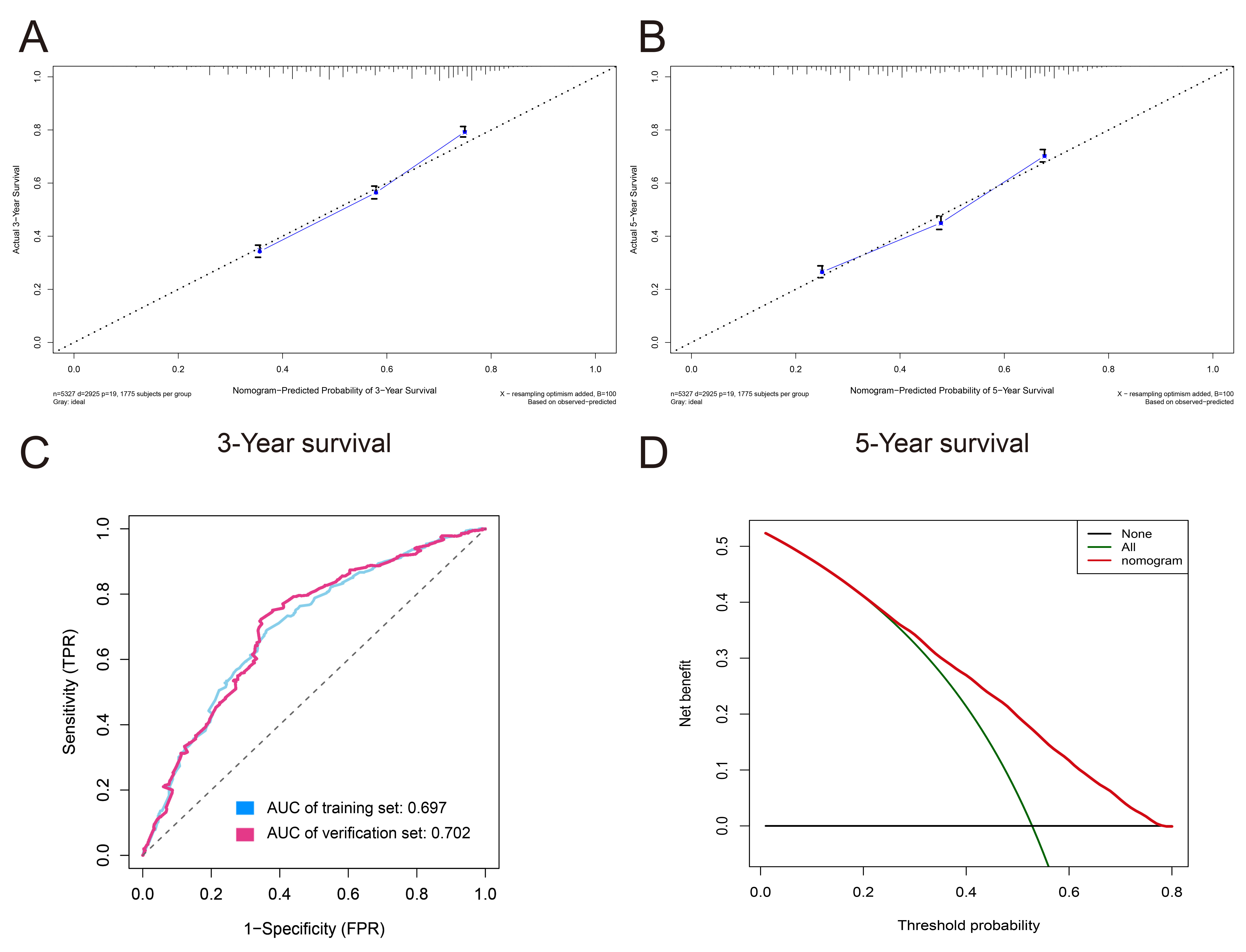

Supplement: Supplementary Figure 2 — Calibration curve, receiver operating characteristic curve, and decision curve analysis (DCA) for predicting overall survival (OS). Calibration curve of the 3- (A) and 5-year (B) OS nomograms in the training set (bootstrap method, 1000 repetitions). (C) Area under the curve for predicting the OS of patients with BCA in the training and validation sets. (D) The DCA curve of the training set. The x-axis represents the threshold probability, and the y-axis represents the net benefit. The black line indicates that all patients are at low risk, whereas the green line indicates that all patients are at high risk. [file Image_2.jpeg]

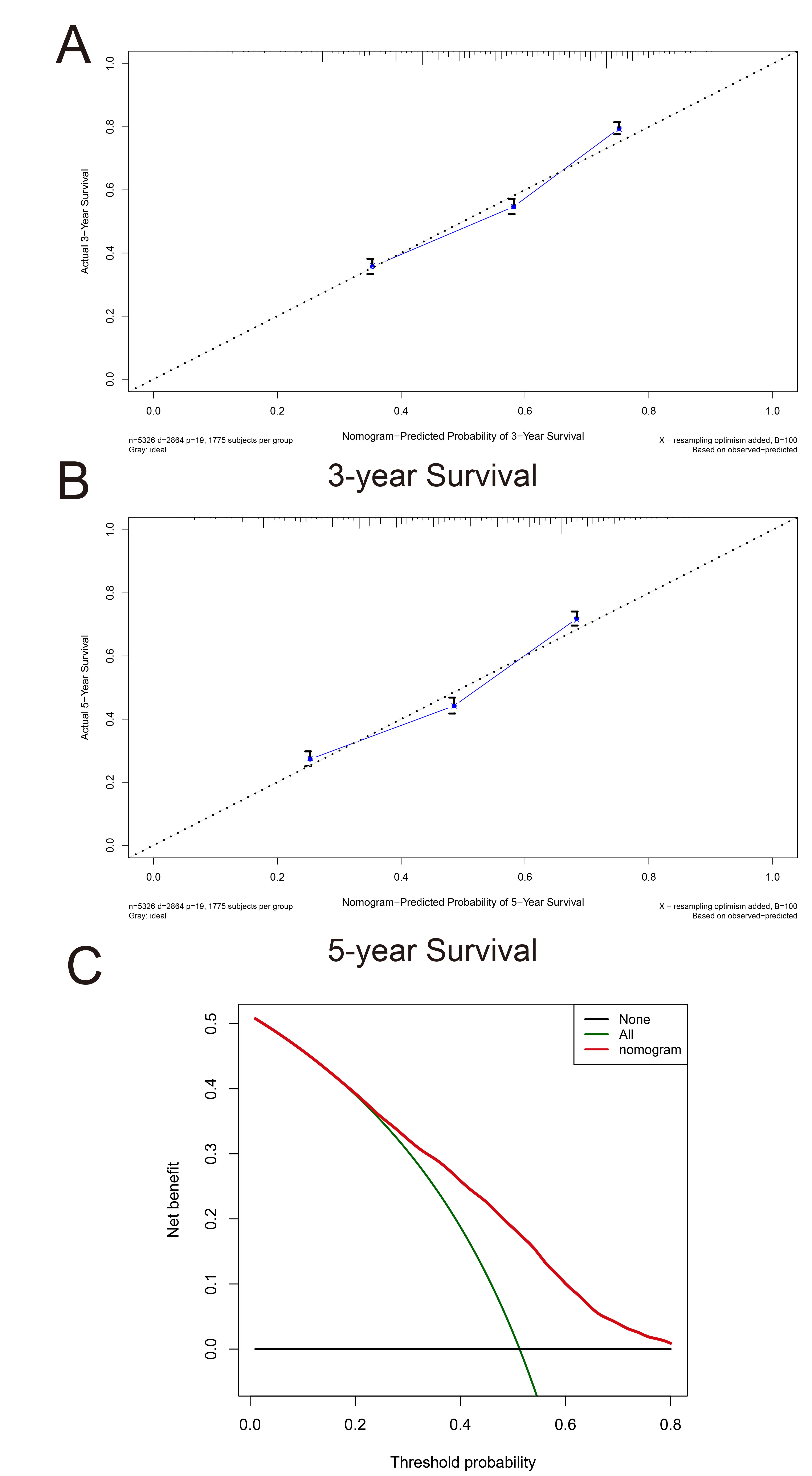

Supplement: Supplementary Figure 3 — Calibration curve and decision curve analysis (DCA) for predicting overall survival in patients with bladder cancer. Calibration curve of the 3- (A) and 5-year (B) overall survival nomograms in the verification set (bootstrap method, 1000 repetitions). (C) The DCA curve of the verification set. The x-axis and y-axis mean the threshold probability and net benefit, respectively. The black line indicates that all patients are at low risk, whereas the green line indicates that all patients are at high risk. [file Image_3.jpeg]
