# Supplementary material for: Predictive Nomogram and Risk Factors for Lymph Node Metastasis in Bladder Cancer
Source: Front Oncol. 2021 Jun 16;11:690324. doi: 10.3389/fonc.2021.690324 (PMC8242250; doi:10.3389/fonc.2021.690324)
Supplement: Supplementary file 4 [file Table_1.docx]

Supplementary. Table S1 Cox regression analysis of the prognostic factors for overall survival

| Clinicopathological variables |  | Univariate analysis |  |  | Multivariate analysis |  |
| --- | --- | --- | --- | --- | --- | --- |
|  |  | OR (95%CI) | P value |  | OR (95%CI) | P value |
| **Age at diagnosis** |  |  |  |  |  |  |
| <50 |  | Reference |  |  | Reference |  |
| 50-65 |  | 1.213 (0.986-1.492) | 0.068 |  | 1.203 (0.978-1.480) | 0.080 |
| 65-79 |  | 1.573 (1.286-1.925) | <0.001 |  | 1.581 (1.291-1.936) | <0.001 |
| >80 |  | 2.477 (2.004-3.062) | <0.001 |  | 2.451 (1.979-3.035) | <0.001 |
| **Sex** |  |  |  |  |  |  |
| Female |  | Reference |  |  |  |  |
| Male |  | 0.926 (0.852-1.006) | 0.071 |  |  |  |
| **Race** |  |  |  |  |  |  |
| Caucasians |  | Reference |  |  | Reference |  |
| Afro-Americans |  | 1.314 (1.142-1.511) | <0.001 |  | 1.216 (1.056-1.401) | 0.007 |
| Other |  | 0.891 (0.755-1.050) | 0.168 |  | 0.875 (0.742-1.032) | 0.112 |
| Unknown |  | 0.201 (0.050-0.802) | 0.023 |  | 0.243 (0.061-0.970) | 0.045 |
| **Grade** |  |  |  |  |  |  |
| Grade I |  | 0.553 (0.397-0.769) | <0.001 |  | 0.953 (0.670-1.355) | 0.788 |
| Grade II |  | 0.911 (0.752-1.104) | 0.343 |  | 1.356 (1.104-1.664) | 0.004 |
| Grade III |  | 1.112 (1.028-1.203) | 0.008 |  | 1.121 (1.036-1.213) | 0.005 |
| Grade IV |  | Reference | < |  | Reference |  |
| **Tumor size** |  |  |  |  |  |  |
| <1cm |  | Reference |  |  | Reference |  |
| 1-2cm |  | 1.349 (1.072-1.696) | 0.011 |  | 1.184 (0.941-1.491) | 0.150 |
| 2-3cm |  | 1.339 (1.079-1.662) | 0.008 |  | 1.064 (0.856-1.322) | 0.577 |
| 3-4cm |  | 1.462 (1.182-1.808) | <0.001 |  | 1.131 (0.913-1.401) | 0.260 |
| 4+cm |  | 1.829 (1.496-2.237) | <0.001 |  | 1.309 (1.068-1.604) | 0.010 |
| **T** |  |  |  |  |  |  |
| T1 |  | Reference |  |  | Reference |  |
| T2 |  | 1.159 (0.983-1.366) | 0.079 |  | 1.118 (0.947-1.320) | 0.189 |
| T3 |  | 2.534 (2.163-2.968) | <0.001 |  | 2.229 (1.895-2.623) | <0.001 |
| T4 |  | 3.557 (3.007-4.207) | <0.001 |  | 3.081 (2.590-3.664) | <0.001 |
| Ta |  | 0.839 (0.628-1.121) | 0.236 |  | 0.760 (0.558-1.034) | 0.080 |
| Tis |  | 1.052 (0.518-2.138) | 0.888 |  | 0.960 (0.468-1.971) | 0.912 |
| **N** |  |  |  |  |  |  |
| N0 |  | Reference |  |  | Reference |  |
| N1 |  | 1.437 (1.335-1.546) | <0.001 |  | 1.217 (1.126-1.314) | <0.001 |

Abbreviations: OR, odd ratio; 95%CI, 95% confidence intervals.
