# Supplementary material for: Predictive Nomogram and Risk Factors for Lymph Node Metastasis in Bladder Cancer
Source: Front Oncol. 2021 Jun 16;11:690324. doi: 10.3389/fonc.2021.690324 (PMC8242250; doi:10.3389/fonc.2021.690324)
Supplement: Supplementary file 5 [file Table_2.docx]

Supplementary. Table S2 Cox regression analysis of overall survival prognostic factors in lymph node non-metastatic bladder cancer

| Clinicopathological variables |  | Univariate analysis |  |  | Multivariate analysis |  |
| --- | --- | --- | --- | --- | --- | --- |
|  |  | OR (95%CI) | P value |  | OR (95%CI) | P value |
| **Age at diagnosis** |  |  |  |  |  |  |
| <50 |  | Reference |  |  | Reference |  |
| 50-65 |  | 1.296 (0.895-1.877) | 0.170 |  | 1.292 (0.892-1.873) | 0.176 |
| 65-79 |  | 1.885 (1.314-2.704) | 0.001 |  | 1.794 (1.250-2.576) | 0.002 |
| >80 |  | 3.382 (2.340-4.888) | <0.001 |  | 3.090 (2.136-4.470) | <0.001 |
| **Sex** |  |  |  |  |  |  |
| Female |  | Reference |  |  |  |  |
| Male |  | 0.983 (0.866-1.115) | 0.789 |  |  |  |
| **Race** |  |  |  |  |  |  |
| Caucasians |  | Reference |  |  | Reference |  |
| Afro-Americans |  | 1.260 (1.012-1.568) | 0.039 |  | 1.171 (0.939-1.461) | 0.161 |
| Other |  | 0.859 (0.674-1.096) | 0.221 |  | 0.866 (0.679-1.105) | 0.248 |
| Unknown |  | 0.343 (0.086-1.374) | 0.131 |  | 0.537 (0.134-2.157) | 0.381 |
| **Grade** |  |  |  |  |  |  |
| Grade I |  | 0.672 (0.474-0.952) | 0.025 |  | 1.007 (0.692-1.466) | 0.971 |
| Grade II |  | 1.025 (0.817-1.285) | 0.834 |  | 1.509 (1.179-1.932) | 0.001 |
| Grade III |  | 1.086 (0.965-1.223) | 0.170 |  | 1.153 (1.023-1.298) | 0.019 |
| Grade IV |  | Reference |  |  | Reference |  |
| **Tumor size** |  |  |  |  |  |  |
| <1cm |  | 0.579 (0.447-0.750) | <0.001 |  | 0.789 (0.606-1.027) | 0.078 |
| 1-2cm |  | 0.716 (0.596-0.859) | <0.001 |  | 0.845 (0.703-1.016) | 0.074 |
| 2-3cm |  | 0.748 (0.642-0.871) | <0.001 |  | 0.821 (0.704-0.958) | 0.012 |
| 3-4cm |  | 0.789 (0.681-0.914) | 0.002 |  | 0.818 (0.705-0.949) | 0.008 |
| 4+cm |  | Reference |  |  | Reference |  |
| **T** |  |  |  |  |  |  |
| T1 |  | Reference |  |  | Reference |  |
| T2 |  | 1.216 (0.997-1.483) | 0.053 |  | 1.192 (0.975-1.456) | 0.086 |
| T3 |  | 2.593 (2.136-3.149) | <0.001 |  | 2.352 (1.928-2.868) | <0.001 |
| T4 |  | 3.371 (2.687-4.230) | <0.001 |  | 3.111 (2.471-3.916) | <0.001 |
| Ta |  | 0.986 (0.724-1.343) | 0.931 |  | 0.832 (0.596-1.161) | 0.279 |
| Tis |  | 1.009 (0.471-2.160) | 0.981 |  | 0.889 (0.410-1.928) | 0.766 |

Abbreviations: OR, odd ratio; 95%CI, 95% confidence intervals.
